# Worsening Heart Failure and Atrial Flutter in a Patient Secondary to Cardiac Resynchronization Therapy Dyssynchrony: A Case Report

**DOI:** 10.7759/cureus.29096

**Published:** 2022-09-12

**Authors:** Zahid Khan, George Besis, Joseph Tomson

**Affiliations:** 1 Acute Medicine, Mid and South Essex NHS Foundation Trust, Southend-on-Sea, GBR; 2 Cardiology and General Medicine, Barking, Havering and Redbridge University Hospitals NHS Trust, London, GBR; 3 Cardiology, Royal Free Hospital, London, GBR

**Keywords:** cardiac resynchronization therapy-defibrillator, atrial flutter with rapid ventricular response, brain natriuretic peptide (bnp), medication non-compliance, shortness of breath (sob), atrial arrhythmia, heart failure with reduced ejection fraction, left ventricular systolic dysfunction, cardiac resynchronization treatment, cardiac resynchronization therapy (crt)

## Abstract

Cardiac resynchronization therapy-defibrillator (CRT-D) and/or cardiac resynchronization therapy-pacemaker (CRT-P) play an important role in improving cardiac synchronization and reducing the risk of ventricular fibrillation arrest (VFA) in patients with severe left ventricular systolic dysfunction (LVSD). Patients with LVSD may notice worsening symptoms when CRT-D or CRT-P is in dyssynchrony. We present a case of 59-year-old patient who presented with worsening shortness of breath (SOB) and progressive exertional dyspnea for the past few weeks accompanied by pink, frothy sputum, occasional urinary incontinence and urge. He was known to have severe LVSD with an ejection fraction of 10% and had CRT-D in situ. Clinical examination revealed bilateral crepitation and normal heart sounds. A chest radiograph showed pulmonary oedema. An electrocardiogram (ECG) showed atrial fibrillation (AF)/flutter with wide QRS complexes. The patient was treated for acute pulmonary oedema and had CRT-D reprogrammed to achieve biventricular synchrony. He was treated with intravenous furosemide and alternate day metolazone initially. He showed significant subjective and objective improvement and was planned for outpatient synchronized intra-device cardioversion. This case is important because patients with severe LVSD with malfunctioning cardiac resynchronization therapy can result in worsening heart failure (HF) leading to higher morbidity and mortality.

## Introduction

Cardiac resynchronization therapy-defibrillator (CRT-D) or cardiac resynchronization therapy-pacemaker (CRT-P) represents a major advancement in both acute and chronic heart failure (HF) therapies as this is the only therapy that can improve both acute and chronic cardiac systolic function by enhancing biventricular synchrony and improves long-term survival [1]. About 50% of patients with dilated cardiomyopathy have conduction delays such as right bundle branch block (RBBB) or left bundle branch block (LBBB), and as a result of this contractile dyssynchrony, there is marked regional heterogeneity of myocardial contraction, with reduced load in early stimulated regions and higher load in regions of late activation [2,3]. Similar changes are observed in the regional blood flow, and displacement of blood from early to late and from late to early sites results in a net decline in ejected stroke volume [4,5]. The main principle of CRT is simultaneous biventricular preexcitation that restores coordinated biventricular contraction and augmenting chamber ejection and works without causing any rise in the myocardial oxygen requirement, indicating improved chamber mechanical efficiency [6]. We present a case of a 59-year-old patient with known severe left ventricular systolic dysfunction (LVSD) and worsening heart failure symptoms. The patient had CRT-D in situ and was having atrial flutter as underlying rhythm. He had device reprogramming and cardioverted to normal sinus rhythm (NSR) without requiring intra-device or external cardioversion and showed significant symptomatic improvement within 24 hours.

## Case presentation

A 59-year-old patient presented with worsening shortness of breath (SOB) and productive cough with pink, frothy sputum for the past three days, and he was not able to lie flat for the past three weeks. He denied any chest pain but was having urgency of micturition and inability to control his bladder. His past medical history (PMH) was significant for severe left ventricular systolic dysfunction (LVSD) with an ejection fraction of 10%, CRT-D in situ, atrial fibrillation (AF)/flutter, gout, obstructive sleep apnea, high body mass index (BMI), known LBBB, hypertension, coronary artery disease, and descending thoracic aortic aneurysm, 41 mm in size, under vascular follow-up. His LVSD was due to a combination of ischemic and non-ischemic nature. His last CRT-D checks showed normal sinus rhythm with narrow QRS complexes, upsloping QRS complexes in V1-V2, and downsloping QRS complexes in lead 1 about six months ago. He used to work as a gym instructor and was non-compliant with his medications for a brief period of about two weeks over two months ago when he tried herbal medications. He, however, claimed to be compliant with his medications since then.

He lived with his wife and kids and was a lifelong non-smoker and social drinker. His fluid intake was under 1.2 liter over the last 2-3 weeks. He was on apixaban for atrial flutter and had a CRT-D device implanted for severely impaired LVSD over a year ago. Physical examination revealed bilateral crepitations up to mid-zones bilaterally, mild bilateral pedal edema, and normal heart sounds. Vital signs showed an irregular heart rate of 117 beats per minute (bpm), blood pressure of 132/92 mmHg, and oxygen saturation of 96% on 3 liters. He was diagnosed with acute pulmonary edema. Laboratory results are shown in Table 1.

**Table 1:** Laboratory results on day 1 and day 10 of admission CRP: C-reactive protein; BNP: brain natriuretic peptide

Test initials	Day 1	Day 10	Reference range
White cell count	7.85 × 10^9^/L	10.61 × 10^9^/L	3.5-11 × 10^9^/L
Neutrophil count	5.79 × 10^9^/L	7.26 × 10^9^/L	1.7-7.5 × 10^9^/L
Hemoglobin	13.9 g/dL	14.1 g/dL	13.5-17 g/dL
Mean cell volume	79 fL	76.1 fL	79-98 fL
CRP	4 mg/L	47 mg/L	0-5 mg/L
Sodium	140 mmol/L	138 mmol/L	135-145 mmol/L
Potassium	4 mmol/L	4 mmol/L	3.5-5.1 mmol/L
Urea	10.4 mmol/L	12.1 mmol/L	2.1-7.1 mmol/L
Creatinine	148 umol/L	158 umol/L	66-112 umol/L
Troponin T	69 ng/L	52 ng/L	<14 ng/L
N-terminal pro-BNP	7,456 ng/L	2,240 ng/L	<300 ng/L

**Figure 1:**
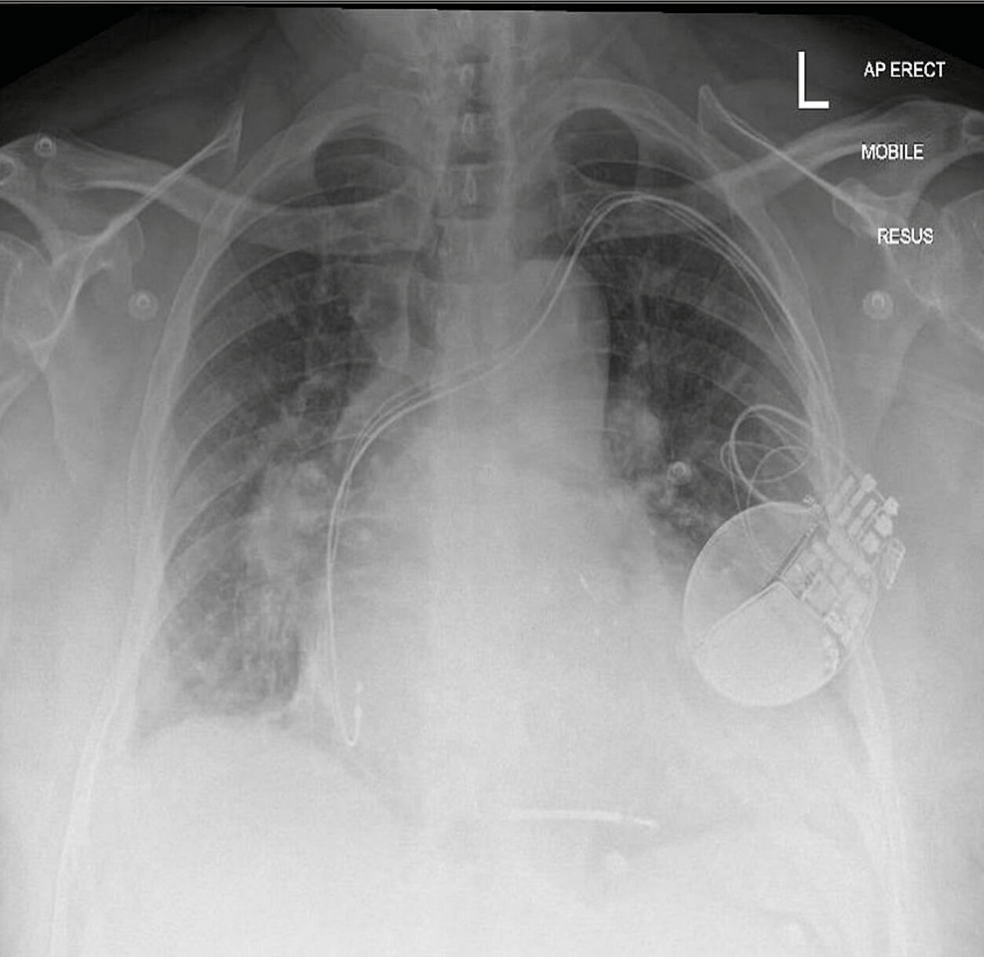
Chest radiography showing pulmonary edema and cardiac resynchronization therapy defibrillator in situ

Chest radiography shows acute pulmonary edema with CRT-D in situ (Figure 1).

**Figure 2:**
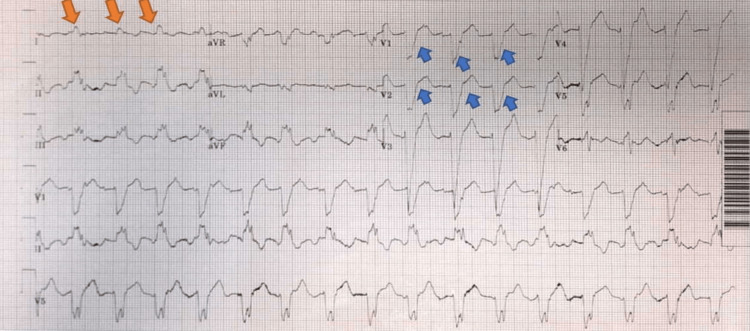
Atrial flutter with left bundle branch block, upsloping QRS in lead 1 (red arrows), and downsloping QRS in V1-V2 (blue arrows)

Electrocardiogram (ECG) showed atrial flutter with wide QRS complexes, upsloping QRS complex in lead 1, and downsloping QRS complexes in V1-V2, consistent with CRT-D dyssynchrony as optimally programmed device generally should have downsloping QRS complexes in leads 1 and upsloping QRS and narrow QRS complexes in leads V1-V2 (Figure 2).

**Figure 3:**
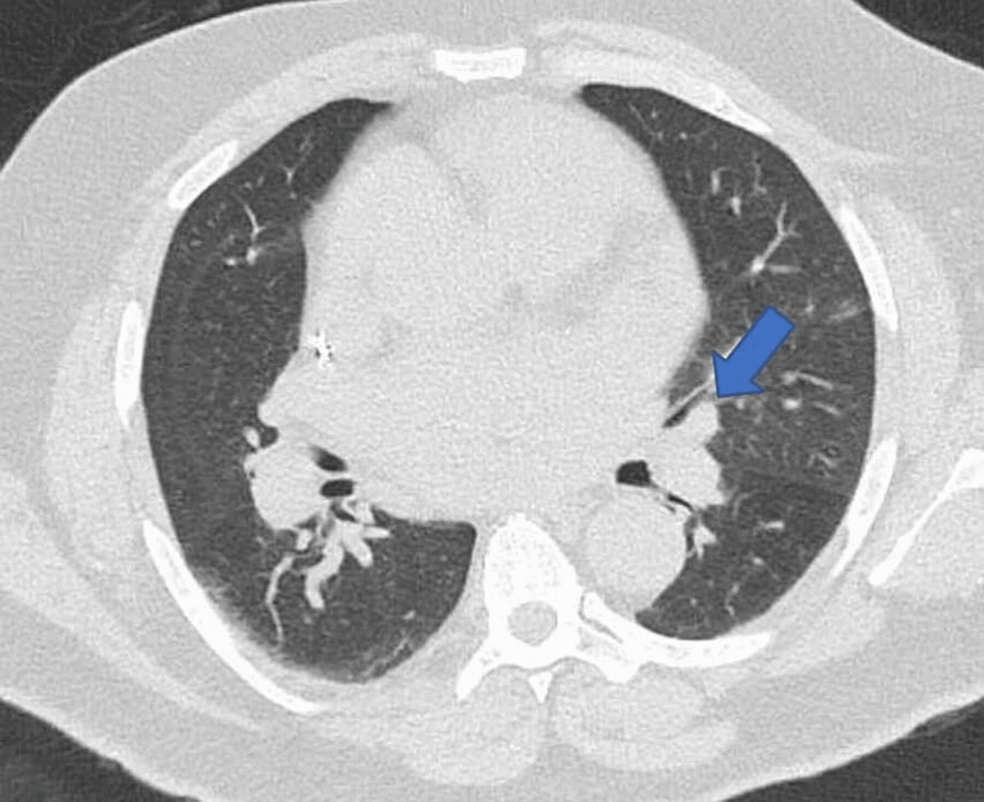
CTPA showing enlarged lingular lymph node (blue arrow) CTPA: computed tomography pulmonary angiogram

**Figure 4:**
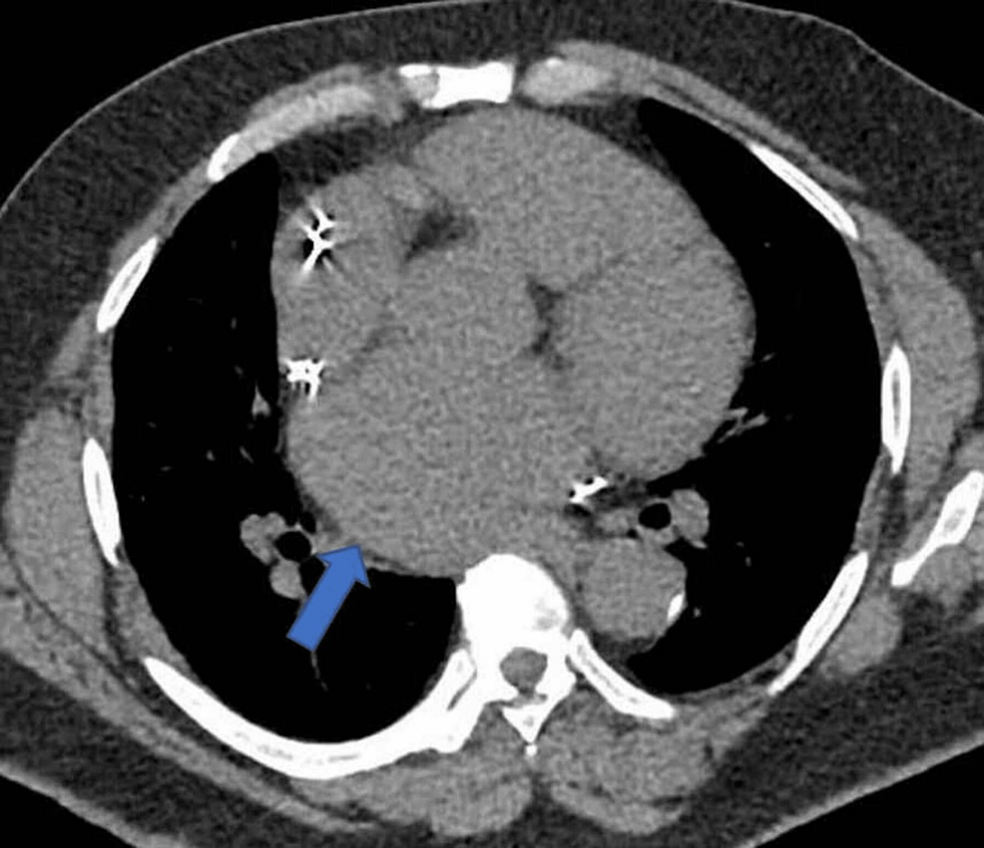
CTPA showing enlarged left atrium (blue arrow) CTPA: computed tomography pulmonary angiogram

A high-resolution computed tomography (HRCT) scan of the thorax showed a 6 mm/48 mm^3^ nodule in the lingula and dilated left atrium. The ascending thoracic aorta and pulmonary artery were dilated, measuring 45 mm and 40 mm, respectively (Figures 3, 4).

An echocardiogram showed severely biventricular dilatation, severely impaired LVSD with an estimated ejection fraction of 8%, and good right ventricular longitudinal systolic function but impaired radial or transverse systolic function and severe biatrial dilatation (Videos 1-3).

**Video 1:** Echocardiogram apical four-chamber view showing biventricular dilatation and impaired function

**Video 2:** Echocardiogram parasternal long-axis view showing impaired dilated left ventricular cavity with an impaired ejection fraction

**Video 3:** Echocardiogram parasternal short-axis view showing an impaired left ventricular function

**Figure 5:**
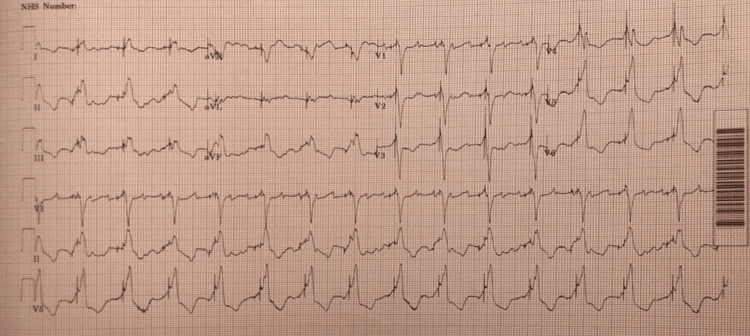
Narrow QRS complexes and atrial flutter immediately after CRT-D reprogramming

**Figure 6:**
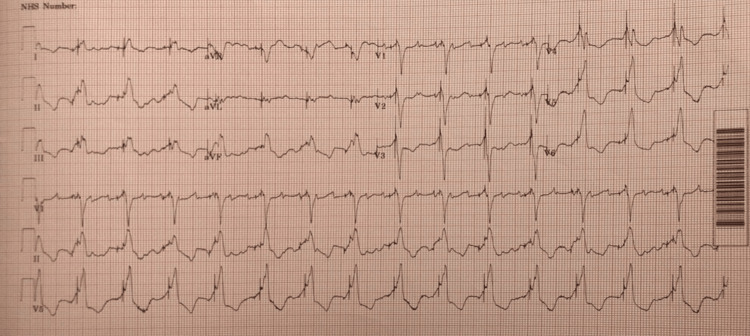
Narrow QRS complexes and atrial flutter immediately after CRT-D reprogramming

CRT-D interrogation revealed underlying atrial flutter with a rapid ventricular response and cardiac dyssynchrony. The patient was commenced on intravenous furosemide 80 mg twice daily, ramipril 5 mg, bisoprolol 10 mg, eplerenone 25 mg once daily, and other regular medications. He lost 5 kg weight and required alternate days of metolazone 2.5 mg doses along with furosemide to offload him. Despite losing weight over a couple of days, he did not show any significant improvement. CRT-D was reprogrammed to achieve biventricular pacing evidenced by smaller QRS complexes with downsloping QRS in lead 1 and upsloping or narrow QRS complexes in lead V1-V2 on day 4, and he remained in atrial flutter for the next two days (Figures 5, 6).

**Figure 7:**
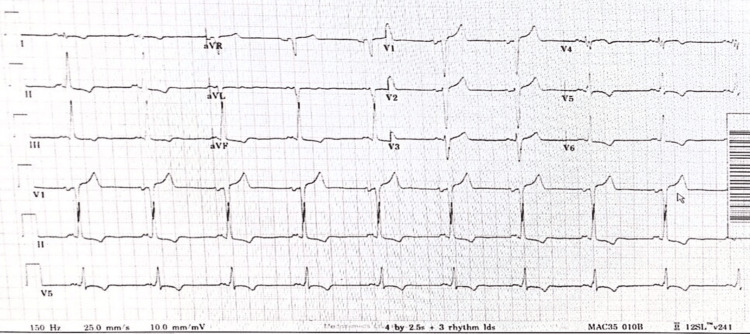
Sinus bradycardia and T wave inversion in infero-lateral leads

LV1-LV2 leads did not result in satisfactory capture; hence, LV3-LV4 leads were attempted for capturing that resulted in satisfactory positive deflection and capture. This is confirmed by a positive vector in V1 and a negative vector in lead 1, which is suggestive of LV activation on the posterolateral capture from CRT and predicts CRT response. In view of recent non-compliance and inability to establish absolute compliance with anticoagulation, intra-device direct current cardioversion (DCCV) was not performed as an inpatient, and the patient was planned for outpatient cardioversion. However, the patient reverted himself to normal sinus rhythm within 48 hours after device reprogramming and hence did not require outpatient cardioversion anymore. The patient showed significant both subjective and objective improvement following his device reprogramming, and his cough and shortness of breath improved significantly within 24 hours of device reprogramming despite being in atrial flutter for the next 24 hours, after which he reverted to NSR with narrow QRS complexes. ECG at the time of discharge showed sinus bradycardia with T wave inversion (TWI) in infero-lateral leads (Figure 7).

He was then commenced on bumetanide 1 mg twice daily and sacubitril/valsartan 24-26 mg twice daily along with other medications. The patient showed significant improvement in his symptoms, and his pedal oedema resolved. Medication compliance was reiterated to the patient, and outpatient follow-up was booked at the pacing clinic in six weeks’ time and the heart failure/arrhythmia clinic in two months’ time. The patient had significantly improved exercise tolerance following the device reprogramming and remained in NSR.

This patient was followed up a month later and showed significant clinical improvement. He remained in sinus rhythm and had improved exercise tolerance.

## Discussion

Cardiac resynchronization therapy improves left ventricular dyssynchrony and contractile function, which is associated with better long-term outcomes [7]. Almost half of heart failure patients die due to arrhythmias, in particular, ventricular fibrillation, and patients with a history of reduced left ventricular ejection fraction (LVEF), previous cardiac arrest, or documented sustained ventricular arrhythmias are, in particular, at high risk [8,9]. The use of implantable cardioverter-defibrillator (ICD) was recommended by both the European Society of Cardiology (ESC) and the American College of Cardiology (ACC) and the American Heart Association (AHA) guidelines in 2005 and by the joint ESC/ACC/AHA guidelines published in 2006 for patients with heart failure.

Several randomized control trials have evaluated over 4,000 heart failure patients with ventricular dyssynchrony on optimal medical therapy alone versus optimal medical therapy plus cardiac resynchronization therapy (pacemaker or defibrillator). Patients with CRT had improved functional status based on the assessment of the quality of life, New York Heart Association (NYHA) Functional Classification, exercise distance during a six-minute walk test, and LVEF in these trials [10-13]. The Comparison of Medical Therapy, Pacing, and Defibrillation in Heart Failure (COMPANION) study and Cardiac Resynchronization - Heart Failure (CARE-HF) trials showed significant mortality and morbidity reduction in patients with CRT-D. In the COMPANION study, CRT reduced all-cause hospital admission by ∼20% and mortality risk by 24%, whereas CRT-D reduced all-cause mortality by 36% [14]. On the other hand, the CARE-HF study showed a 37% all-cause mortality risk reduction from hospital admission or unplanned hospital admission from a major cardiovascular event [15]. In the CARE-HF trial, CRT showed a 52% reduction in hospital admission for patients with heart failure, and CRT alone without a defibrillator showed a 36% reduced risk of death from any cause [15].

The Multicenter Automatic Defibrillator Implantation Trial with Cardiac Resynchronization Therapy (MADIT-CRT) trial showed improved survival benefits in patients with heart failure who had CRT-D fitted in comparison to ICD alone or only medical therapy. The study involved 761 patients with New York Heart Association (NYHA) class I/II, ejection fraction of ≤30%, and QRS of ≥130 ms [16]. From this group, 434 patients had CRT-D, 327 patients had ICD, and all these patients had echocardiograms at baseline and in 12 months’ time. A greater improvement in LV dyssynchrony and contractile function was demonstrated in the CRT-D group compared with the ICD-only group. The trial also showed that there was a significant reduction in one-year mortality and heart failure after improvement in dyssynchrony and contractile function after adjusting for baseline characteristics and that each 20 ms decrease in LV dyssynchrony was associated with a 7% reduction in the primary outcome. Similarly, each 1% improvement in global longitudinal strain (GLS) over the 12-month period was associated with a 24% reduction in mortality or heart failure [16].

Our patient had CRT-D, which was lacking synchronization as evident from the wide QRS complexes, upsloping QRS in lead 1, and downsloping QRS in V1, and the patient showed significant improvement after resynchronization of the CRT-D. The patient also reverted back to normal sinus rhythm (NSR) three days following device reprogramming. Haugaa et al. reported that patients with persistent or new dyssynchrony after CRT-D implantation in patients with severe heart failure symptoms, widened QRS complexes, and reduced left ventricular ejection fraction were associated with increased mortality and had a less favorable prognosis [17].

The European Society of Cardiology (ESC) recommends CRT in symptomatic patients with heart failure who are in normal sinus rhythm and with LV ejection fraction (LVEF) of ≤35%, QRS duration of ≥150 ms, and left bundle branch block (LBBB) QRS morphology [18]. It also recommends the consideration of CRT in symptomatic patients with HF in sinus rhythm with LVEF of ≤35%, QRS duration of 130-149 ms, and LBBB QRS morphology. In addition, CRT is also recommended in patients with HF who are in sinus rhythm and have LVEF of ≤35%, QRS duration of ≥150 ms, and non-LBBB QRS morphology. The 2021 ESC guidelines also recommend that patients with HF and LVEF of ≤35%, NYHA class III or IV who are in atrial fibrillation, and intrinsic QRS of ≥130 ms should also be considered for CRT implantation, provided that biventricular capture can be achieved, and patients with incomplete biventricular pacing (<90%-95%) due to the conducted AF should also have atrioventricular (AV) junction ablation [18].

## Conclusions

In conclusion, CRT-D resynchronization is associated with improved patient survival, reduced rate of hospital admission, and reduced mortality risk due to arrhythmias, in particular, ventricular fibrillation. The strength of this study lies in the fact that clinical trials have shown the benefits of cardiac resynchronization before, and our patient also showed significant improvement following successful device reprogramming. It is important to interrogate devices in patients with clinical deterioration in their heart failure symptoms despite having CRT-D in situ.
